# A Rare Case of Lipidized Fibrous Histiocytoma of the Oral Cavity

**DOI:** 10.7759/cureus.82265

**Published:** 2025-04-14

**Authors:** Katsumitsu Shimada, Satoshi Murakami, Hiromasa Hasegawa

**Affiliations:** 1 Department of Clinical Pathophysiology, Matsumoto Dental University, Shiojiri, JPN; 2 Hard Tissue Pathology Unit, Graduate School of Oral Medicine, Matsumoto Dental University, Shiojiri, JPN; 3 Department of Oral Pathology/Forensic Odontology, School of Dentistry, Aichi Gakuin University, Nagoya, JPN

**Keywords:** cd68, factor xiiia, foam cells, lipidized fibrous histiocytoma, oral cavity

## Abstract

Benign fibrous histiocytoma (FH) is a common cutaneous tumor that rarely occurs in the oral cavity. Lipidized FH is an uncommon variant characterized by abundant foam cells in collagenized stroma. To our knowledge, only a single case of oral lipidized FH affecting the tongue has been reported. We examine a case of lipidized FH in the buccal mucosa of a patient presenting with a gradually enlarging, pedunculated lesion measuring up to 26 mm. A histological examination revealed a well-marginated but unencapsulated tumor with a Grenz zone, hyalinized stroma, and central foam cell aggregation. Immunohistochemically, the spindle and foam cells were CD68-positive, with focal Factor XIIIa positivity and negative bcl-2 staining. The lesion was completely excised, and no recurrence was observed. To our knowledge, this is the second reported case of oral lipidized FH and the first case in the buccal mucosa. Given its rarity and histological overlap with other xanthomatous lesions, accurate diagnosis is crucial with immunohistochemistry. Complete excision appears to be curative; however, a long-term follow-up is recommended, considering the subtype of fibrous histiocytoma developing in the orofacial region.

## Introduction

Dermatofibroma (fibrous histiocytoma) is rarely found in the oral cavity. However, this tumor is the most common cutaneous mesenchymal neoplasm according to the World Health Organization (WHO) classification of tumors [1]. A recent systematic review identified 59 cases of oral benign fibrous histiocytoma (FH) using available anatomical site data. Although the cheek is the site most frequently affected by benign FH, only 12 cases (20%) were reported, followed by the mandible and tongue [2].

Benign FH exhibits significant histological diversity depending on its stage, appearing more cellular in early lesions and more fibrotic with fewer macrophages in older lesions [1]. Numerous histological subtypes have been found, including fibro-collagenous, lipidized, clear cell, granular cell, halo, osteoclastic, myofibroblastic, keloidal, palisading, atrophic, and signet ring cell variants [3-5]. Given this diversity, the diagnosis of benign FH can be challenging [3-5].

Lipidized FH is a rare subtype, accounting for 2% of all cases [5] and usually presents as a solitary, exophytic yellow nodule. Histologically, the lesion is characterized by the accumulation of numerous foam cells within a background of hyalinized, wiry, keloid-like, and occasionally osteoid-like collagen bundles [4]. Initial studies suggested a predilection for the lower extremities, particularly around the ankles [6]. Cases involving the oral cavity remain exceedingly rare. 

To date, only one case of lipidized FH affecting the tongue has been reported [7]. Here, we present an additional example of lipidized FH arising from the buccal mucosa with detailed histological and immunohistochemical findings.

## Case presentation

The patient is a 39-year-old female who presented to the Department of Oral and Maxillofacial Surgery with a gradually growing tumor in her right cheek. The patient had no remarkable family or medical history, such as bite wounds. Laboratory test results revealed no abnormalities.

**Figure 1:**
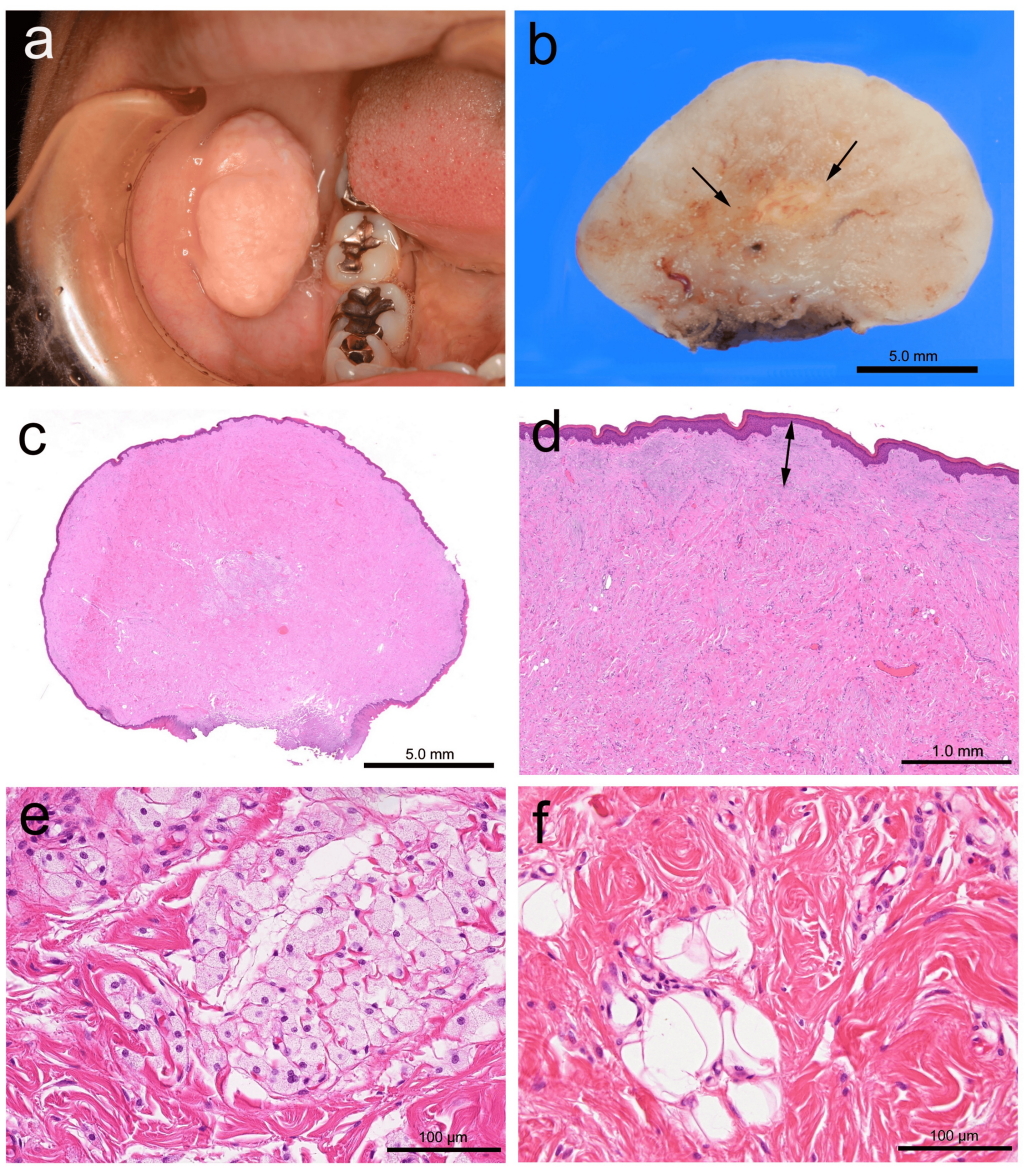
Macroscopic and histological findings An exophytic tumor mass on the cheek (a) with a cut surface colored light tan and a yellowish area (arrows) in the center (b). Histologically, the pedunculated mass is entirely fibrous (c) with a subepithelial Grenz zone (double arrow) (d). Aggregation of foam cells (e) and scattered spindle cells with bland nuclei, and mature fat cells embedded in the collagenous stroma (f). The scale bars represent 5 mm (b and c), 1 mm (d), and 100 μm (e and f), respectively.

Intraoral examination revealed a large pedunculated tumor on the right cheek, exhibiting an elastic-hard consistency. The overlying epithelium appeared almost normal but slightly white in part, and had a somewhat irregular, granular surface (Figure 1a). Clinically, the lesion was diagnosed as fibroma. The excised specimen measured 26 × 15 mm, and the cut surface was light tan in color, with a yellowish area in the center (Figure 1b). Five years after the 2019 surgery, no signs of recurrence or metastasis have been noted.

**Figure 2:**
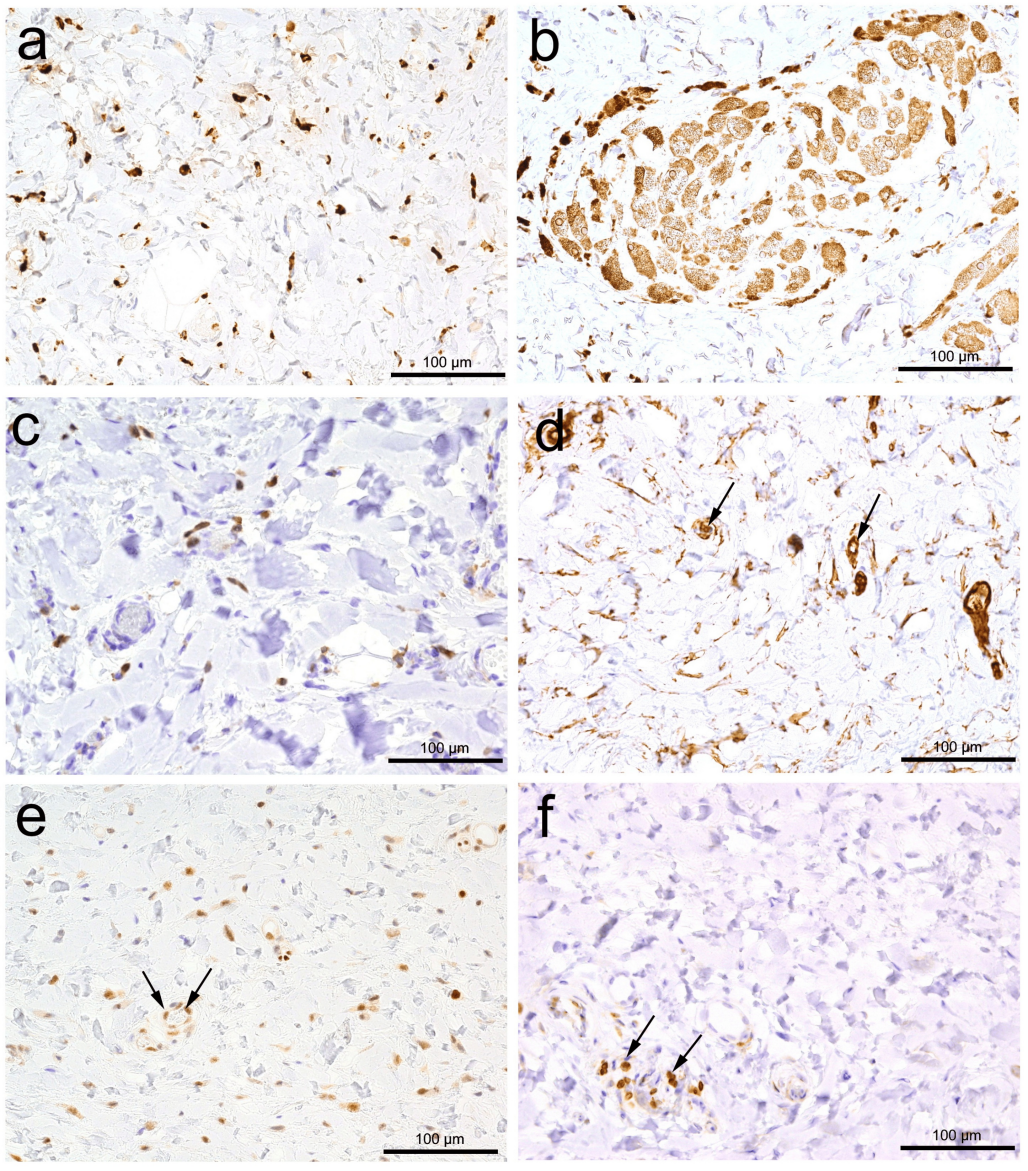
Immunohistochemical findings Immunohistochemical analysis showed CD68 positivity in both spindle cells (a) and foam cells (b). Spindle cells were positive for Factor XIIIa (c), CD34 (d), and Rb (e), but negative for bcl-2 (f). Additionally, note the positive reactions of capillaries (arrows), endothelial cell nuclei (arrows), and lymphocytes (arrows) for CD34 (d), Rb (e), and bcl-2 (f), respectively. All scale bars represent 100 µm.

Microscopic examination revealed a pedunculated exophytic lesion covered by a stratified squamous epithelium. The fibrous nodule was completely excised, with a free margin from the tumor (Figure 1c). The lesion was unencapsulated but relatively well-marginated, with a Grenz zone (an area of intervening normal connective tissue) separating it from the overlying epithelium, which exhibited hyperorthokeratosis. The lesion predominantly consisted of deeply eosinophilic hyalinized stroma containing haphazardly arranged thick collagen bundles (Figure 1d). In the central portion, there was a distinct aggregation of cohesively arranged foam cells along with a few adipose cells embedded in the hyalinized stroma. The nuclei of both scattered spindle and foam cells were bland, and no mitotic figures were observed (Figures 1e and 1f). Immunohistochemical examination revealed CD68-positive spindle and foam cells. Additionally, some spindle cells were positive for Factor XIIIa but negative for bcl-2. Rb protein was completely retained in spindle cells (Figure 2). Based on these histological and immunohistochemical findings, a final diagnosis of lipidized FH was made.

## Discussion

We describe a rare case report of lipidized FH in the buccal mucosa. The histological and immunohistochemical features of this case were consistent with lipidized FH: a rare variant of fibrous histiocytoma characterized by the presence of foam cells within a collagenized stroma. The well-marginated but unencapsulated nature of the lesion along with the presence of a Grenz zone is a recognized feature of cutaneous fibrous histiocytomas [4]. The central aggregation of foam cells, accompanied by scattered spindle cells with hyalinized stroma, may lead to diagnostic confusion with other histiocytic or xanthomatous lesions, characterized by the aggregation of foamy macrophages.

Benign FH predominantly occurs in females, and affects individuals with a mean age of approximately 36 years. It most commonly involves the extremities and rarely appears in the head and neck region [8]. Topographically, the cheek is the second most frequent site of benign facial FH [9]. A recent systematic review revealed that benign FH most frequently affects the buccal mucosa, accounting for 20% of all oral benign FH cases [2]. However, the actual frequency of buccal mucosa involvement might be estimated to be lower because some cases that have been listed as involving the buccal mucosa include subcutaneous cases [10, 11] or cases in the buccal space [12]. 

Subcutaneous cases should be considered as cutaneous FH or dermatofibroma. Additionally, the buccal space is bounded medially by the buccinator muscle, the superficial layer of the deep cervical fascia, and the facial expression muscles. There are numerous tumors in this anatomical space. These not only include salivary gland tumors but also mesenchymal tumors originating from muscular, neural, connective, and lymphatic tissues [13]. Therefore, benign FH arising from the buccal space should be classified as deep benign FH.

The differential diagnoses for this case may be established based on the presence of pedunculated mucosal elevation, highly collagenized stroma, bland spindle cells, scattered adipocytes, aggregated foam cells, and immunohistochemical findings with focal expression of CD34, CD68, and Factor XIIIa, as summarized in Table 1.

**Table 1:** Summary of differential diagnosis of lipidized fibrous histiocytoma FH: fibrous histiocytoma; SCL: spindle cell lipoma; Rb: retinoblastoma protein; SMA: smooth muscle actin; +: positive; -: negative

Tumor	Hyalinized collagen	Foam cells	Additional Features	Immuno-histochemistry
Lipidized FH	Present	Present	Spindle cell proliferation	CD34± /CD163+ /CD68+ / Factor XIIIa+
Sclerosing low-fat SCL	Present	Absent	Spindle cell proliferation	CD34+ / Rb loss/ CD163- /CD68-
Desmoplastic fibroblastoma	Present	Absent	Spindle cell proliferation	SMA+ / CD34- / CD163- /CD68-
Sclerotic fibroma	Present	Absent	Plywood-like pattern	CD34+ / CD163- /CD68-
Verrucous xanthoma	Absent	Present	Epithelial hyperplasia	CD163+ /CD68+
Juvenile xanthogranuloma	Absent	Present	non-Langerhans cell histiocytic proliferation	CD163+ /CD68+ / Factor XIIIa+
Plexiform xanthomatous tumor	Absent	Present	Plexiform pattern	CD163+ /CD68+ / Factor XIIIa+

The observation of mature adipocytes within a hyalinized stroma resembles a sclerosing low-fat spindle cell lipoma, which has been secondarily infiltrated by foam cells. Spindle cell lipomas infrequently present with low-fat or fat-free histological variants, which poses diagnostic challenges [14]. This lesion typically shows diffuse and strong immunoreactivity for CD34. Diffuse and strong CD34 positivity is characteristics of this lesion. Additionally, sclerotic lipomas are composed of cytologically bland spindle cells that lack CD34 expression, embedded in dense fibrosclerotic stroma, and with randomly dispersed adipocytes as a minor component [15]. However, they lack scattered fibrohistiocytic cells, and accumulated foam cells. While the presence of CD34-positive spindle cells is reminiscent of low-fat spindle cell lipoma, some benign FH may also express CD34 focal [16], which does not support the spindle cell lipoma diagnosis. In addition, the absence of bcl-2 protein and the complete retention of Rb protein in this case also represents a lack of characteristic features of spindle cell lipoma [17,18].

Desmoplastic fibroblastoma (collagenous fibroma) and sclerotic fibroma should be considered in the differential diagnosis because they involve hyalinized collagen fibers. However, both lack foam cell aggregation, which distinguishes them from lipidized fibrous histiocytomas. Additionally, spindle cells of desmoplastic fibroblastoma focally express smooth muscle actin but not CD34 [19]. In contrast, the spindle cells of sclerotic fibromas are positive for CD34. Hyalinized thick collagen bundles of sclerotic fibroma characteristically arrange in a plywood-like or whorled pattern, which serves as another diagnostic clue [20,21].

Several types of histiocytic lesions with foam cells can be observed in the oral cavity. Verruciform xanthoma is the most frequent tumor affecting the oral mucosa and is characterized by the aggregation of foam cells and verrucous hyperplasia of the overlying epithelium [22], which can be easily differentiated from the current case.

In other non-Langerhans histiocytic disorders, foam cells can arise in the oral cavity. Juvenile xanthogranuloma (JXG) is a rare, benign proliferative non-Langerhans cell histiocytic proliferation that most often presents in children with cutaneous-flesh colored, yellow-orange, brown, or purple macules or papules. The lesion is entirely occupied by large vacuolated cells with small round or indented nuclei but it lacks a prominent fibrous component. Additionally, these cells were positive for CD163, CD68, and Factor XIIIa, but negative for CD34 [23].

A plexiform xanthomatous tumor (PXT) was described by Michal in 1994 [24] as a fibrohistiocytic neoplasm with prominent foam cells. It shares morphological and immunohistochemical features with xanthomas, cholesteric FH, and lipidized FH. However, its characteristic "plexiform architecture" differentiates PXT from the current case [25].

Benign FHs show many histological variants, including lipidized, hemosiderotic, keloidal, granular cell, palisading, atrophic, clear cell, myxoid, lichenoid, balloon cell, signet-ring cell, aneurysmal, and epithelioid variants [4]. Among these, lipidized FH was first reported by Iwata and Fletcher as a variant of FH [6], also known as the "ankle type." Histologically, it is characterized by the accumulation of numerous foam cells against a background of hyalinized, wiry (thin and wavy), and keloid-like collagenous bundles [4,6]. These characteristics are consistent with the findings in the present case.

The recurrence rate following incomplete excision is estimated to be <5% [1]. Lipidized FH of skin had not documented recurrence or metastasis after complete resection [6]. However, from an anatomical point of view, facial dermatofibromas, in particular, have a higher tendency (22%, 95% CI: 6-26%) to recur than those at other sites because of the difficulty in achieving complete excision [26]. A systematic review revealed that local recurrence was found in 3 of 37 patients, accounting for 8% of cases in the oral-maxillofacial region [2]. Considering these facts, benign FHs in the orofacial region tend to show a higher tendency to recur. Therefore, lipidized FH, a subtype of benign FH in the orofacial region, may also recur after incomplete resection, while this case might have a low risk of recurrence because of a tumor-free margin. Cutaneous "benign" FH can rarely metastasize to the lungs or lymph nodes, with primary lesions lacking morphologic features that predict an adverse outcome and exhibiting similar morphology in the metastases [27]. However, a study has suggested the risk factors for the development of metastases in cutaneous FH are relatively large size, high cellularity, aneurysmal changes, marked cellular pleomorphism, high mitotic activity, tumor necrosis, and repeated local recurrences [28]. Although this case was relatively large, it lacked other risk factors, including the histological features. Therefore, our case appears to have no risk of metastasis. However, FH has no reliable clinical or histologic predictors of prognosis, and its metastasis has been reported to occur up to 15 years after initial diagnosis [27]. In view of these points, long-term follow-up is necessary.

## Conclusions

This case represents the second reported instance of oral lipidized FH that affected the buccal mucosa. Given the rarity of this variant in the oral cavity and its potential for histological misdiagnosis, awareness of its distinct features is essential. Complete excision appears to be curative with a low risk of recurrence. However, a long-term follow-up is recommended due to the potential for late recurrence or metastasis in rare cases.
